# Fermentative Polyhydroxybutyrate Production from a Novel Feedstock Derived from Bakery Waste

**DOI:** 10.1155/2014/819474

**Published:** 2014-07-20

**Authors:** Daniel Pleissner, Wan Chi Lam, Wei Han, Kin Yan Lau, Lai Chun Cheung, Ming Wui Lee, Ho Man Lei, Kin Yu Lo, Wai Yee Ng, Zheng Sun, Mehmet Melikoglu, Carol Sze Ki Lin

**Affiliations:** ^1^School of Energy and Environment, City University of Hong Kong, Tat Chee Avenue, Kowloon, Hong Kong; ^2^Department of Chemical and Biomolecular Engineering, Hong Kong University of Science and Technology, Clear Water Bay, Kowloon, Hong Kong; ^3^College of Fisheries and Life Science, Shanghai Ocean University, Shanghai 201306, China; ^4^Department of Chemical Engineering, Gebze Institute of Technology, 41400 Gebze, Kocaeli, Turkey

## Abstract

In this study, *Halomonas boliviensis* was cultivated on bakery waste hydrolysate and seawater in batch and fed-batch cultures for polyhydroxybutyrate (PHB) production. Results demonstrated that bakery waste hydrolysate and seawater could be efficiently utilized by *Halomonas boliviensis* while PHB contents between 10 and 30% (w/w) were obtained. Furthermore, three methods for bakery waste hydrolysis were investigated for feedstock preparation. These include: (1) use of crude enzyme extracts from *Aspergillus awamori*, (2) *Aspergillus awamori* solid mashes, and (3) commercial glucoamylase. In the first method, the resultant free amino nitrogen (FAN) concentration in hydrolysates was 150 and 250 mg L^−1^ after 20 hours at enzyme-to-solid ratios of 6.9 and 13.1 U g^−1^, respectively. In both cases, the final glucose concentration was around 130–150 g L^−1^. In the second method, the resultant FAN and glucose concentrations were 250 mg L^−1^ and 150 g L^−1^, respectively. In the third method, highest glucose and lowest FAN concentrations of 170–200 g L^−1^ and 100 mg L^−1^, respectively, were obtained in hydrolysates after only 5 hours. The present work has generated promising information contributing to the sustainable production of bioplastic using bakery waste hydrolysate.

## 1. Introduction

The natural polymer polyhydroxybutyrate (PHB) belongs to the group of polyhydroxyalkanoates and is synthesized by over 75 different genera of bacteria as intracellular carbon and energy storage compound [1]. Similar chemical properties to polypropylene [2] and its biodegradability make PHB a potential sustainable alternative to petroleum-based plastics. Moreover, PHB is nontoxic and highly biocompatible with mammalian cells, tissues, and organs and, thus, applicable as biomaterial in tissue engineering for surgical implants and scaffolds, biodegradable screws and staples, and wound dressings [3]. Consequently, much effort has been made to produce PHB at industrial scale in an economical and feasible way. Several bacterial strains such as* Alcaligenes latus*,* Azotobacter vinelandii*,* Cupriavidus necator* (formerly classified as* Wautersia eutropha*), and recombinant* Escherichia coli* were investigated as PHB producers with high productivities [4–12]. Furthermore,* Halomonas boliviensis, *a bacterium which is able to accumulate more than 80% (w/w) of its biomass as PHB under high salt conditions, was investigated [9]. However, fermentative PHB production in large-scale with these bacteria is cost-inefficient, as glucose, commonly used as carbon source, accounts for approximately 30% of the total production costs [13].

In Hong Kong, over 3,500 tonnes of industrial, commercial, and domestic food wastes are generated daily [14, 15]. These food wastes are rich in starch and proteins, which could serve as a source of glucose and amino acids after hydrolysis. Our group previously has demonstrated that food waste hydrolysate could serve as a nutrient source to various microorganisms [15–18]. Therefore, utilization of organic wastes as nutrient, particularly as carbon sources, could be an option to reduce fermentation costs. Also, it would contribute to the development of an economically feasible industrial PHB production. Furthermore, in order to create a cost-efficient fermentation medium, Lin et al. [19] reported the use of seawater as a natural mineral and sustainable water source. Recently, three hydrolytic approaches for the recovery of nutrients from food waste have been reported, which include (1) the use of commercial glucoamylases [20, 21]; (2) solid fungal mashes of* Aspergillus awamori* and/or* Aspergillus oryzae* [15–17, 22]; and (3) crude enzyme extract from* Aspergillus awamori* and/or* Aspergillus oryzae *[18, 23, 24]. However, there is lack of systematic studies concerning the efficiency of hydrolytic approaches for the recovery of nutrients from food waste.

In this study, we aim to provide an adaptation strategy on methods that could lead to efficient production of fermentation feedstock in presence of seawater for sustainable production of PHB. Firstly, hydrolyzed bakery waste and seawater were investigated as fermentation feedstock and medium, respectively, for PHB production in batch and fed-batch cultures of* Halomonas boliviensis*. Secondly, the efficiency of glucose and free amino nitrogen (FAN) productions from bakery waste using three hydrolytic approaches: (1) crude enzyme extract from* Aspergillus awamori*, (2) commercial glucoamylase, and (3)* Aspergillus awamori* solid mashes were compared.

## 2. Materials and Methods

### 2.1. Handling of Bakery Waste

The material used for the production of hydrolysate was out-of-date bakery waste (pastry and cake) obtained from a Starbucks store located in Shatin, Hong Kong. Bakery waste was homogenized immediately after collection by a kitchen blender and stored at 4°C.

### 2.2. Microorganisms

*Aspergillus awamori* (ATCC 14331) was purchased from the American Type Culture Collection (Rockville, MD, USA) and used for fungal solid mashes and crude enzyme extract production.

Spore suspension of* Aspergillus awamori* was prepared by spore extraction with 10% (w/v) glycerol from a culture grown for 7 days at 30°C on 1.5% (w/v) corn meal agar. Counting of spores was carried out using a haemocytometer.

*Halomonas boliviensis *BAA-759 was purchased from the American Type Culture Collection (Rockville, MD, USA) and stored in cryopreservation vials at −80°C until used in fermentation. Inocula for fermentative PHB production were grown in 250 mL shake flasks containing 100 mL defined medium consisting of 10 g L^−1^ glucose, 2 g L^−1^ yeast extract, 40 g L^−1^ NaCl, 0.75 g L^−1^ KCl, 0.38 g L^−1^  MgSO_4_ × 7H_2_O, 0.2 g L^−1^ NaBr, and 0.13 g L^−1^  CaCl_2_ × 2H_2_O for 24 hours at 35°C and an initial pH value of 7.5 [25]. Shake flasks were agitated at 200 rpm by an orbital shaker.

### 2.3. Mineral Concentration of Seawater

Mineral concentration was determined after evaporation of a known volume of seawater.

### 2.4. Fermentative Polyhydroxybutyrate Production

Fermentative PHB production by* Halomonas boliviensis* in batch and fed-batch cultures was performed using bakery waste hydrolysate at 35°C and pH 7.5. The pH was automatically controlled by the addition of 2 M NaOH and 2 M H_2_SO_4_. Stirring speed was set between 850 and 1,300 rpm in order to maintain dissolved oxygen above 20%. Antifoam was added manually when needed. A 2% (v/v) inoculum was used in all fermentations. Samples for quantification of glucose, fructose, FAN, and biomass concentrations as well as weight specific PHB content were taken regularly, centrifuged at 11,000 ×g for 10 minutes, and stored at −80°C. Bakery waste hydrolysate used in batch cultures was prepared as described by Pleissner et al. [16] in seawater with a mineral concentration of 29.7 g L^−1^. Bakery waste hydrolysate used in fed-batch cultures was concentrated by water evaporation using a rotary evaporator.

### 2.5. Quantification of Glucose, Fructose, and Free Amino Nitrogen

Glucose and fructose were analyzed using high performance liquid chromatography (Waters, UK) by injection of 10 *μ*L of supernatant on an Aminex HPX-87H column (Bio-Rad, USA) and eluted isocratically with 5 mM H_2_SO_4_ at 0.5 mL min^−1^ and 65°C. Detection was performed by a refractive index detector (Waters, UK) at 35°C.

Free amino nitrogen (FAN) concentration was measured based on the ninhydrin reaction method as described by Lie [26]. Glycine was used as reference compound.

### 2.6. Biomass Concentration

Biomass concentration of* Halomonas boliviensis* was measured as cell dry weight after centrifugation of a known volume of culture broth at 11,000 ×g for 10 minutes and lyophilization of the pellet.

### 2.7. Polyhydroxybutyrate Extraction, Esterification, and Quantification

Extraction and esterification of PHB from freeze-dried biomass of* Halomonas boliviensis* was carried out according to the method of Riis and Mai [27]. To 0.02 g of freeze dried biomass, 2 mL dichloroethane, 2 mL acidified propanol (propanol : HCl, 4 : 1, v/v), and 0.2 mL benzoic acid were added and the mixture was incubated for two hours at 100°C. After chilling to room temperature, 4 mL of demineralised water was added and the suspension was mixed. The organic phase was recovered after mixing.

Quantification of extracted PHB was performed by injection of 1 *μ*L of the recovered organic phase on an Agilent PoraPLOT Q-HT (USA) column connected to a gas chromatography system (Hewlett Packard 6890 GC System, USA). Detection was carried out using a flame ionization detector with helium as carrier gas. Following temperature programme was used: initial temperature 90°C held for 2 minutes then increased to 150°C at a rate of 8°C minute^−1^ and held for 4 minutes and finally increased to 280°C at 20°C minute^−1^ and held for 1 minute. The retention time and peak area were compared to standards (PHB and benzoic acid) of known concentration.

### 2.8. *Aspergillus awamori* Solid Mashes

For preparation of* Aspergillus awamori* solid mashes, 10–15 g of wet bakery waste (7–10.5 g dry weight) was placed in a petri dish and inoculated with 5 × 10^5^ spores per gram wet waste. The fungal mashes were incubated for 7 to 8 days at 30°C.

### 2.9. Crude Enzyme Extract

Crude enzyme extract from* Aspergillus awamori* was prepared by homogenizing 7 to 8 days old fungal solid mashes from three petri dishes thoroughly in a kitchen blender containing 30 mL demineralized water. Afterwards, additional 30 mL demineralized water was added into the blender and the mixture was stirred at 30°C for 30 minutes followed by centrifugation at 5,500 ×g for 10 minutes. Crude enzyme extract refers to the obtained supernatant after filtration with Whitman number 1 filter paper.

### 2.10. Commercial Glucoamylase

Commercial glucoamylase (Glucose Amylase) was provided by Shandong Longda Bio-products, Co., Ltd., China.

### 2.11. Glycolytic Activity Assay

Glycolytic activity of the crude enzyme extract from* Aspergillus awamori* and commercial glucoamylase was determined using the method described previously [23]. One unit (U) of glycolytic activity is defined as the amount of enzyme that releases 1 *μ*mol of glucose per minute at pH 5.5 and 55°C.

### 2.12. Proteolytic Activity Assay

Proteolytic activity of the crude enzyme extract was quantified using a modified version of the method described by Wang et al. [28] based on the formation of FAN after hydrolyzing 0.6% (w/v) casein solubilized in 0.2 M potassium phosphate buffer (pH 7.5). Enzymatic hydrolysis was performed by adding 0.25 mL of crude enzyme extract to 0.5 mL casein solution. The reaction mixture was then incubated at 55°C for 30 minutes and terminated by adding 0.25 mL of 10% (w/v) trichloroacetic acid (TCA). The concentration of FAN released from casein was quantified based on nynhidrin reaction method described in Section 2.5. One unit of protease activity (U) was defined as the production of 1 *μ*g FAN by 1 mL crude enzyme extract in one minute under reaction conditions.

### 2.13. Investigation of Bakery Waste Hydrolysis

Hydrolysis of 548.8 g (dry weight) homogenized bakery waste was conducted with one of the following methods: (1) crude enzyme extract from* Aspergillus awamori* solid mashes from three petri dishes; (2) commercial glucoamylase added in a ratio of 6.9 and 13.1 U per gram dry bakery waste; and (3)* Aspergillus awamori* solid mashes from three petri dishes in demineralized water. Different enzyme-to-solid ratios were used to investigate the opportunity to adjust glucose and FAN concentrations in hydrolysates. Hydrolysis was carried out at 55°C and pH 4–4.5. A solid-to-liquid ratio, defined as the ratio of dry weight (g) to liquid (mL) [22], of 38 to 47% (w/v) was used in all hydrolyses. In addition, a control experiment without addition of enzymes or fungal biomass was carried out under the same experimental conditions. Samples of 1.2 mL were taken at regular intervals and immediately mixed with 350 *μ*L 10% (w/v) TCA in order to deactivate enzymes and to prevent further hydrolysis. Quantification of glucose and FAN in supernatant was performed after centrifugation of the sample-TCA mixture at 11,000 ×g for 10 minutes.

Independently, seawater was investigated as medium in hydrolysis. To 330 g (dry weight) homogenized bakery waste, 1 U g^−1^ of a commercial glucoamylase was added and the hydrolysis was carried out at 55°C and pH 4–4.5 in seawater with a mineral concentration of 29.7 g L^−1^.

### 2.14. Composition of Bakery Waste

Quantification of carbohydrate, nitrogen, and protein contents in bakery waste was performed in duplicate as described in Pleissner et al. [16]. All specific contents of waste constituents reported in this study are based on dry weights. Quantification of soluble and insoluble fractions of carbohydrates, nitrogen, and proteins was carried out in a control experiment described in Section 2.13. The soluble fraction of carbohydrates, nitrogen, and proteins in bakery waste refers to the fraction that was solubilized in water, while the insoluble fraction of each waste constituent refers to the fraction that was not soluble in water.

## 3. Results and Discussion

### 3.1. Fermentative Polyhydroxybutyrate Production

**Figure 1:**
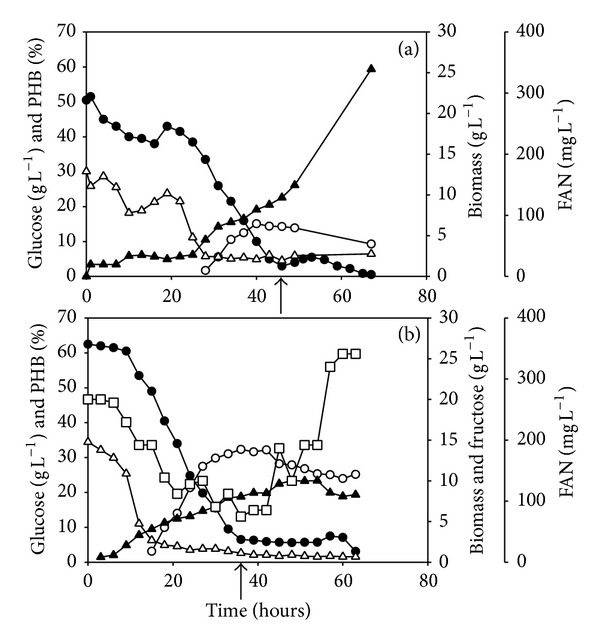
Fermentative PHB production. Profile of glucose (closed circle), fructose (open square), free amino nitrogen (FAN, open triangle), and biomass (closed triangle) concentrations as well as weight specific PHB content (open circle) during fermentation of* Halomonas boliviensis* on bakery waste hydrolysate ((a) and (b)). Fermentations were initially performed as batch cultures and later changed to fed-batch cultures. Start of feeding is indicated by an arrow.

The feasibility of using bakery waste hydrolysate and seawater as medium for fermentative PHB production with* Halomonas boliviensis* was examined.  Figure 1(a) shows the fermentation of* Halomonas boliviensis* on hydrolyzed bakery waste and seawater at initial glucose and FAN concentrations of 50.5 g L^−1^ and 172.2 mg L^−1^, respectively. After 30 hours, the FAN concentration was reduced to 32 mg L^−1^ and the PHB content increased to 14% (w/w) due to nitrogen limitation of* Halomonas boliviensis* cells. When glucose concentration became below 5 g L^−1^, a hydrolysate consisting of 103.0 g L^−1^ glucose and 606.2 mg L^−1^ FAN was fed continuously at a rate of 0.029 L h^−1^ for 20 hours in order to prevent glucose depletion. During fed-batch phase, cells consumed all the glucose (59.7 g) and FAN (351.6 mg) supplied. The feeding resulted in a further increase in biomass concentration from 5 to 25 g L^−1^ due to the supply of glucose and FAN in excess. The PHB content of cells, however, remained unchanged at 10–14% (Table 1).

A second fermentation was conducted similar to the first fermentation. The FAN concentration of the hydrolysate used throughout fed-batch phase, however, was much lower in order to limit biomass formation and to facilitate PHB accumulation (Figure 1(b)). For the initial batch phase, a cake rich bakery waste hydrolysate consisting of 62.5 g L^−1^, 20.0 g L^−1^, and 197.2 mg L^−1^ glucose, fructose, and FAN, respectively, was used (Figure 1(b)). FAN was rapidly consumed within 20 hours and the PHB concentration consequently increased to 32.3% (w/w) between 20 and 30 hours of fermentation. During batch phase, around 10 g L^−1^ biomass was formed and a simultaneous consumption of glucose and fructose was observed. After 38 hours, when glucose concentration was below 5 g L^−1^, a cake rich bakery waste hydrolysate consisting of 67.0 g L^−1^ glucose and 29.6 g L^−1^ fructose, but only 79.9 mg L^−1^ FAN was fed continuously at a rate of 0.034 L h^−1^ for 24 hours. During fed-batch phase, cells consumed around 54.7 g glucose and 65.2 mg FAN. In comparison to the first fermentation, the use of a hydrolysate with a lower FAN concentration limited the formation of biomass. However, the PHB content was not further increased during fed-batch phase and 25.2 mg g^−1^ PHB was found after continuous feeding (Figure 1(b) and Table 1). Fructose, as an additional carbon source, was not consumed during fed-batch and its concentration increased to more than 25 g L^−1^.

Generally, the molar yield of PHB was two times higher in fed-batch cultures compared to batch cultures, while the PHB content did not change (Figure 1 and Table 1). Based on the data reported for batch cultures of* Halomonas boliviensis* using defined media and glucose [29], and glucose and xylose [11] as carbon sources, yields of 0.13 and 0.24 mol PHB mol^−1^ carbon substrate, respectively, were calculated. These yields are comparable to the yields (0.16 and 0.17 mol PHB mol^−1^ carbon substrate, Table 1) obtained in batch cultures in this study using food waste hydrolysate. The conversion yield during fed-batch phase of the first fermentation was 0.23 mol PHB mol^−1^ carbon substrate (Table 1 and Figure 1(a)) which is similar to the yield obtained in batch phase due to the formation of biomass. However, in the second fermentation when the amount of nitrogen in feed hydrolysate was reduced, formation of biomass was limited while the molar yield of PHB increased to 0.33 mol PHB mol^−1^ carbon substrate. This yield is considerably higher than the yield found in batch phase (0.17 mol PHB mol^−1^ carbon substrate, Table 1) and the yield, 0.13 mol PHB mol^−1^ carbon substrate, obtained by Quillaguamán et al. [9] in fed-batch cultures using a defined medium containing glucose as carbon source.

Results from fermentations show that* Halomonas boliviensis* could grow in the presence of seawater and utilize nutrients from bakery waste hydrolysate. This suggests that hydrolysate and seawater formed a nutrient complete medium for PHB production.

### 3.2. Investigation of Bakery Waste Hydrolysis

Fed-batch cultures are the most popular culture system to achieve high PHB contents. Fed-batch cultures are usually split into two stages: batch phase for cell growth and fed-batch phase for PHB accumulation. In* Halomonas boliviensis* fermentation, the medium used in batch phase should be rich in glucose and FAN in order to facilitate high cell concentration. On the other hand, medium used in fed-batch phase should be rich in glucose but depleted in FAN for PHB accumulation (Figure 1) [30, 31]. Furthermore, the medium used in fed-batch phase should be sufficiently concentrated to minimize the dilution effect due to increased volume of fermentation broth. In order to investigate which hydrolytic approaches would lead to the production of hydrolysates with targeted nutrient concentrations, bakery waste was hydrolyzed using the three methods explained in Section 2.13. The bakery waste used in experiments consisted of 720.4 mg g^−1^ total carbohydrates, 22.7 mg g^−1^ total nitrogen, and 129.5 mg g^−1^ total proteins, and is appropriate for the production of a hydrolysate that is rich in glucose and/or FAN (Table 2).

**Figure 2:**
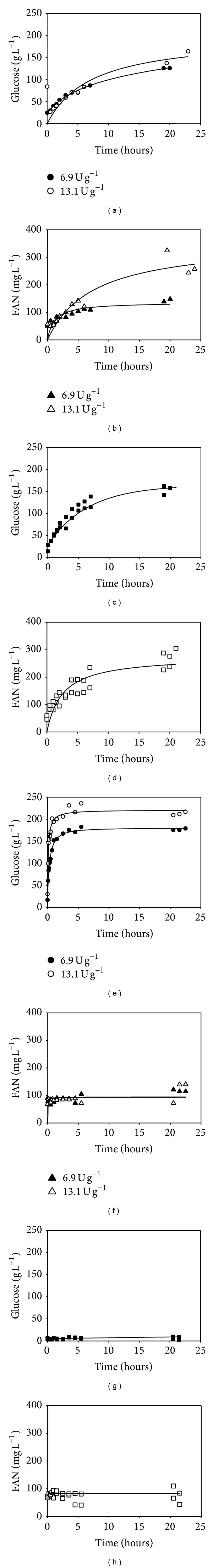
Change in glucose and FAN concentrations in hydrolysate during bakery waste hydrolysis using a crude enzyme extract from* Aspergillus awamori* solid mashes ((a) and (b)),* Aspergillus awamori* solid mashes directly added ((c) and (d)), commercial glucoamylase ((e) and (f)), and in a control ((g) and (h)), respectively. Hydrolyses carried out using crude enzyme extract and commercial glucoamylase were performed at enzyme-to-solid ratios of 6.9 and 13.1 U g^−1^.

Firstly, bakery waste hydrolysis was carried out using crude enzyme extracts from* Aspergillus awamori* solid mashes at enzyme-to-solid ratios of 6.9 and 13.1 U g^−1^. In both cases, similar glucose concentrations of 130–150 g L^−1^ were found in hydrolysates after 20 hours (Figure 2(a)). However, the resultant FAN concentrations showed significant difference (Table 3, Figure 2(b)). When hydrolysis was performed at 6.9 U g^−1^, 150 mg L^−1^ of FAN was obtained. When the enzyme-to-solid ratio was increased to 13.1 U g^−1^ by addition of a larger volume of crude enzyme extract, the FAN concentration also increased to 250 mg L^−1^.* Aspergillus awamori* is known for the secretion of both glycolytic and to a lesser extent of proteolytic enzymes [22]. Therefore, the addition of a larger volume of the crude enzyme extract led not only to increased initial glycolytic activity, but also proteolytic activity (data not shown) and consequently to higher FAN concentration in hydrolysate. This finding highlighted the possibility to adjust the resultant FAN concentration using crude enzyme extract in order to prepare a fermentation feedstock which is favourable for promoting biomass formation, while the glucose concentration remains unaffected. Figures 2(c) and 2(d) show the glucose and FAN production profiles when hydrolysis was carried out using* Aspergillus awamori* solid mashes. The final glucose and FAN concentrations were similar to the concentrations obtained after hydrolysis of bakery waste carried out at an enzyme-to-solid ratio of 13.1 U g^−1^ (Figures 2(a) and 2(b)). Thus, there is no need to extract enzymes from fungal solid mashes prior to the use in hydrolysis.

Figure 2(e) shows glucose production profiles when hydrolyses were carried out using commercial glucoamylase at enzyme-to-solid ratios of 6.9 and 13.1 U g^−1^. At 13.1 U g^−1^ the resultant glucose concentration and yield were 200 g L^−1^ and 0.39 g g^−1^, respectively, while around 170 g L^−1^ glucose and a slightly lower yield of 0.32 g g^−1^ were obtained at 6.9 U g^−1^. The addition of commercial glucoamylase did not only result in highest glucose concentrations (200 g L^−1^), but it also reached complete hydrolysis in less than 5 hours. This highly efficient hydrolytic performance leads to a reduced process period and eventually decreases the operating cost. The FAN concentration in the hydrolysate was low (around 100 mg L^−1^), as no additional FAN was released from bakery waste as compared to the control (Table 3, Figures 2(f) and 2(h)). Microbial contaminations introduced by unsterile bakery waste did not contribute to the degradation of carbohydrates and proteins, as only trace amounts of glucose (<20 g L^−1^) and FAN (<80 mg L^−1^) were found in the control supernatant (Figures 2(g) and 2(h)). Instead, the presence of trace amounts of glucose and FAN probably originated from the water soluble carbohydrate and nitrogen fractions from bakery waste (Table 2). The fact that microbial contaminations neither degrade carbohydrates and proteins nor metabolize sugars and FAN contributes to a more controllable fermentation medium preparation process, which enables the production of a nutrient-complete fermentation feedstock with the targeted nutritional compositions and values. A medium consisting of 200 g L^−1^ glucose, but only 100 mg L^−1^ FAN would facilitate PHB accumulation during fed-batch phase. At the same time, due to its high glucose concentration, the dilution effect due to increasing fermentation broth volume during fed-batch cultivation can be minimized.

Apart from glucose, fructose was found in hydrolysates by the hydrolysis of sucrose in cake rich bakery wastes [15]. Although the yields of fructose were 7 to 10 times lower compared to the yields of glucose (Table 3), it was utilized by* Halomonas boliviensis* and contributed to cell growth and PHB formation (Figure 1(b)).

Table 3 shows that similar yields of glucose, fructose, and FAN were obtained when hydrolysis was carried out using commercial glucoamylase in seawater as solvent instead of tap water. Domínguez de María [32] reviewed the development of a seawater-based biorefinery strategy for enzymatic, fermentative, and chemocatalytic applications. In combination with food waste-based biorefinery, the use of seawater may have a strong impact on the development of efficient, low cost, and small carbon footprint PHB production processes [32].

## 4. Conclusions

In this study, bakery waste hydrolysate was evaluated as nutrient source in fermentative PHB production with seawater by* Halomonas boliviensis*. Moreover, the feasibility of using seawater as solvent in bakery waste hydrolysis for fermentative feedstock preparation was demonstrated. Bakery waste hydrolysates can be utilized together with seawater as fermentation media in batch and fed-batch cultures. By selecting an appropriate hydrolytic approach of bakery waste, the concentration of glucose and FAN could be adjusted towards biomass or PHB formation in* Halomonas boliviensis* fermentation. The fed-batch fermentation strategies tested did improve the yield of PHB, but the PHB content of cells was not affected. Nevertheless, the encouraging results suggested that a generic feedstock from bakery waste forms a nutrient-complete medium for microbial growth of* Halomonas boliviensis* and pave the way for a sustainable bioplastic production.

## Figures and Tables

**Table 1:** PHB fermentations by *Halomonas boliviensis* using bakery waste hydrolysate and defined medium using glucose and xylose as carbon sources.

Medium	Process	PHB content [%, w/w]	PHB concentration [g L^−1^]	Yield of PHB [mol PHB mol^−1^ carbon substrate]	References
Bakery waste hydrolysate	Batch	13.7	1.1	0.16	This study
	Fed-batch	9.3	2.4	0.23	
	Batch	32.3	2.6	0.17	
	Fed-batch	25.2	2.1	0.33	

Glucose	Fed-batch	81	35.4	0.13	[9]

Glucose/xylose	Batch	44.9 ± 0.41	1.0	0.24	[11]

Glucose	Batch	55	0.5	0.13	[29]

**Table 2:** Composition of bakery waste.

Food waste constituent	Weight specific content [mg g^−1^]
Total carbohydrates	720.4
Soluble carbohydrates	134.4
Insoluble carbohydrates	586.0
Total nitrogen	22.7
Soluble nitrogen	5.4
Insoluble nitrogen	17.4
Total proteins	129.5
Soluble proteins	30.5
Insoluble proteins	99.0

**Table 3:** Yield of glucose (*Y*_Glc_), fructose (*Y*_Frc_), and free amino nitrogen (*Y*_FAN_) obtained by different hydrolytic treatments of bakery waste.

Treatment	Seawater	Enzyme-to-solid ratio [U g^−1^]	*Y*_Glc_ [g g^−1^]	*Y*_Frc_ [g g^−1^]	*Y*_FAN_ [mg g^−1^]
Multienzyme solution	No	6.9	0.23	0.02	0.29
		13.1	0.29	0.04	0.41

Commercial glucoamylase	No	6.9	0.32	0.03	0.22
		13.1	0.39	0.04	0.26

Commercial glucoamylase	Yes	1.0	0.28	0.09	0.36

Control	No	—	0.01	0.01	0.19
